# Reversal of profound vecuronium-induced neuromuscular block under sevoflurane anesthesia: sugammadex versus neostigmine. 

**DOI:** 10.1186/1471-2253-10-15

**Published:** 2010-09-01

**Authors:** Hendrikus JM Lemmens, Mohammad I El-Orbany, James Berry, Jovino Ben Morte, Gavin Martin

**Affiliations:** 1 Stanford University Medical Center, Stanford, CA, USA; 2 Advocate Illinois Masonic Medical Center, Chicago, IL, US; 3 Vanderbilt University Medical Center, Nashville, TN, USA; 4 Merck Research Laboratories, Summit, New Jersey, USA; 5 Duke University Medical Center, Durham, NC, USA; 6 The Medical College of Wisconsin, Milwaukee, WI, USA

## Abstract

**Background:**

Acetylcholinesterase inhibitors cannot rapidly reverse profound neuromuscular block. Sugammadex, a selective relaxant binding agent, reverses the effects of rocuronium and vecuronium by encapsulation. This study assessed the efficacy of sugammadex compared with neostigmine in reversal of profound vecuronium-induced neuromuscular block under sevoflurane anesthesia.

**Methods:**

Patients aged ≥18 years, American Society of Anesthesiologists class 1-4, scheduled to undergo surgery under general anesthesia were enrolled in this phase III, multicenter, randomized, safety-assessor blinded study. Sevoflurane anesthetized patients received vecuronium 0.1 mg/kg for intubation, with maintenance doses of 0.015 mg/kg as required. Patients were randomized to receive sugammadex 4 mg/kg or neostigmine 70 μg/kg with glycopyrrolate 14 μg/kg at 1-2 post-tetanic counts. The primary efficacy variable was time from start of study drug administration to recovery of the train-of-four ratio to 0.9. Safety assessments included physical examination, laboratory data, vital signs, and adverse events.

**Results:**

Eighty three patients were included in the intent-to-treat population (sugammadex, n = 47; neostigmine, n = 36). Geometric mean time to recovery of the train-of-four ratio to 0.9 was 15-fold faster with sugammadex (4.5 minutes) compared with neostigmine (66.2 minutes; p < 0.0001) (median, 3.3 minutes with sugammadex versus 49.9 minutes with neostigmine). No serious drug-related adverse events occurred in either group.

**Conclusions:**

Recovery from profound vecuronium-induced block is significantly faster with sugammadex, compared with neostigmine. Neostigmine did not rapidly reverse profound neuromuscular block (Trial registration number: NCT00473694).

## Background

In some types of surgery, including many ENT, thoracic, abdominal and neurosurgical procedures, performed under general anesthesia, maintenance of profound relaxation throughout the procedure may be beneficial. However, anesthesiologists are limited in their capacity to provide such levels of neuromuscular block towards the end of surgery in particular, because the traditional agents available for the reversal of neuromuscular block (acetylcholinesterase inhibitors) cannot rapidly reverse profound block [1]. This is especially important if volatile anesthetics have been used as they are known to potentiate the blocking effect of the neuromuscular blocking agents and impair reversal by the acetylcholinesterase inhibitors [2].

Sugammadex is a new selective relaxant binding agent. Previously, sugammadex has been shown to effectively reverse profound block induced by rocuronium 0.6-1.2 mg/kg, with times from administration of sugammadex to a train-of-four (TOF) ratio >0.9 in the region of 2-3 minutes [3-5]. Vecuronium, a non-depolarizing neuromuscular blocking agent with a greater potency than rocuronium [6], has the same basic steroid nucleus structure as rocuronium. To date, a single dose-finding study has examined the efficacy of sugammadex to reverse profound block induced by vecuronium [3].

The present phase III study was the first to examine the efficacy and safety of sugammadex compared with neostigmine in the reversal of profound neuromuscular block induced by vecuronium. This study consisted of two arms, and also assessed the efficacy and safety of sugammadex compared with neostigmine in the reversal of profound rocuronium block; the two parts of the study were separately powered. Here, we describe the results for the vecuronium arm only and results for the rocuronium arm have been reported separately [5].

## Methods

This was a multicenter, randomized, parallel-group, safety-assessor blinded, phase IIIA trial, designated the Signal study (NCT00473694). Patients were allocated to one of four treatment groups in sequence of their enrollment (rocuronium or vecuronium neuromuscular blocking agent and sugammadex or neostigmine for reversal) using a computer-generated randomization schedule prepared centrally by the study sponsor. Only the safety assessor was blinded to study treatment. Thus, drugs were prepared by an investigator who was not involved in the safety assessments.

### Patients

Adults aged ≥18 years, American Society of Anesthesiologists class 1-4 who were scheduled to undergo elective surgery in the supine position under general anesthesia requiring the use of a neuromuscular blocking agent for tracheal intubation and maintenance of neuromuscular block were eligible for enrollment in this study. Patients were excluded if they had a neuromuscular disorder; a history of malignant hyperthermia; significant renal dysfunction; an allergy to narcotics, muscle relaxants, or other medication used during general anesthesia; were using medication known to interfere with neuromuscular blocking agents (e.g., antibiotics, anticonvulsants, and magnesium); or were pregnant, breast feeding, or of childbearing potential and not using an adequate method of contraception.

The study protocol was approved by the Independent Ethics Committee of each trial center and the study was conducted in compliance with the current revision of the Declaration of Helsinki, the International Conference on Harmonization guidelines, Good Clinical Practice, and current regulatory requirements. All patients provided written informed consent.

### Anesthesia and neuromuscular block

Anesthesia was induced with an intravenous opioid and intravenous propofol, and maintained with an intravenous opioid and sevoflurane. The recommended sevoflurane concentration was <1.5 times the age-adjusted minimum alveolar concentration at the time of sugammadex or neostigmine administration. A single intravenous bolus dose of vecuronium 0.1 mg/kg was administered within 10 seconds into a fast-running venous infusion. Neuromuscular block was maintained with 0.015 mg/kg doses of vecuronium as required.

Neuromuscular function was monitored by acceleromyography at the adductor pollicis muscle using the TOF-Watch^® ^SX (Organon Ireland Ltd, a division of Merck and Co, Inc, Swords, Co. Dublin, Ireland). After induction of anesthesia, the TOF-Watch^® ^SX was affixed to the arm that had the intravenous cannula for drug administration, and stabilized and calibrated in the operating room. The investigators used a standard method to set up and monitor responses using the TOF-Watch^® ^SX, in order to minimize trial center variability.

Repetitive TOF stimulation was applied every 15 seconds at the ulnar nerve until the end of anesthesia or at least until recovery of the TOF ratio to 0.9. Neuromuscular data were collected via a transducer fixed to the volar aspect of the distal phalanx of the thumb. When the first twitch response from the TOF stimulation mode had disappeared after the intubation dose, post-tetanic count (PTC) stimulation was started by delivering a 5-sec, 50 Hz tetanic stimulation, followed after by a 3-sec pause, by applying stimulations at a frequency of 1 Hz for 15 sec and the cycle repeated every 6 min until a PTC of 1 or 2 was achieved. The TOF-Watch^® ^SX automatically prevented the use of the PTC button for 2 minutes after a previous PTC. When considered appropriate by the attending anesthesiologist, spontaneous recovery of neuromuscular function was permitted after the last dose of vecuronium until a target of 1-2 PTC was reached, at which point patients were reversed with either a single bolus dose of sugammadex 4 mg/kg or neostigmine 70 μg/kg with glycopyrrolate 14 μg/kg. Neuromuscular monitoring was switched to the TOF mode as described above and continued until the end of anesthesia at least until recovery of the TOF ratio to 0.9, considered sufficient for safe extubation.

Peripheral body temperature was measured continuously by a thermister at the thenar eminence of the palm, and was kept constant at 32°C during the neuromuscular monitoring process. Central body temperature was maintained continuously at ≥35°C.

Patients were not permitted to receive any muscle relaxant other than vecuronium, or a second dose of sugammadex or any other reversal agent other than neostigmine during the monitoring of neuromuscular transmission. If further muscle relaxation was required after the administration of sugammadex, a non-steroidal muscle relaxant could be administered.

### Assessment of safety

Patients were assessed for consciousness after admission to the recovery room; if they were fully awake and oriented and considered cooperative, 5-second head-lift and general muscular-weakness tests were performed by a blinded safety assessor. Tests were repeated every 15 minutes until the patient could successfully perform the 5-second head-lift and again before transfer from the recovery room. The assessment of general muscle weakness was based on a scale from 0-10, with 0 representing total paralysis, 1 signifying extreme impairment, 9 for close to no impairment, and 10 for normal muscle strength. Scores of 3, 4, 5, etc. denoted increasing muscle strength in approximately 10% increments [5].

Patients who had not reached a TOF ratio of 0.9 before transfer to the recovery room remained intubated, sedated, and ventilated until recovery to a TOF ratio of 0.9, and patients with any evidence of residual neuromuscular block, such as respiratory symptoms, after extubation were maintained with adequate ventilation, using measures such as correction of electrolyte abnormalities, keeping the subject warm, airway support including supplemental oxygen, and prompt review of possible etiologic factors contributing to prolonged neuromuscular block. Patients were also assessed for evidence of reoccurrence of block after initial recovery either by continued monitoring of the TOF trace (looking for a decrease in the TOF ratio to < 0.8) in patients who had not awakened or by presence of respiratory symptoms.

Adequacy of spontaneous ventilation, measured by pulse oximetry and respiratory rate, was assessed for at least 60 minutes after recovery of the TOF ratio to 0.9. Blood samples were collected before the administration of vecuronium, 4-6 hours after reversal agent administration, and at the post-anesthetic visit for biochemistry and full blood count. Urine samples were collected 24 hours before surgery and at the post-anesthetic visit. A physical examination was performed by the blinded safety assessor at screening and at the post-anesthetic visit. Vital signs (blood pressure and heart rate) were assessed at screening, before the administration of vecuronium, before the administration of study drug, at 2, 5, and 10 minutes after, and at the post-anesthetic visit.

All adverse events (AEs), serious AEs, laboratory data, and vital signs were recorded for the safety analysis. AEs were assessed by a safety assessor blinded to treatment randomization. All AEs and serious AEs were coded using MedDRA (International Federation of Pharmaceutical Manufacturers and Associations, Chantilly, Virginia, US) version 9.1.

### Statistical analysis

The standard deviation of time to recovery of the TOF ratio to 0.9 was assumed to be 1.5 minutes with sugammadex and 7.0 minutes with neostigmine. Based on this, a sample size of 30 patients per group would have a 95% probability of detecting a difference of 5 minutes in the time to recovery of the TOF ratio to 0.9. Assuming a 5% drop-out rate, 32 patients per group would be needed. In order to distribute enrollment evenly over nine trial sites, the intended sample size was 36 patients per group. The statistical analyses performed in this study have been reported previously for the rocuronium arm of the study [5].

### Efficacy analyses

Data are presented as geometric means and medians with overall and interquartile ranges. The primary efficacy variable was time from start of administration of sugammadex or neostigmine to recovery of the TOF ratio to 0.9. A two-way analysis of variance was used to detect the treatment effect, the center effect and the interaction effect. If an interaction effect was found to exist, the treatment effect for each center was examined individually. If no interaction effect was present, an additive model (without interaction) effect was analyzed. Secondary efficacy variables included the time from the start of administration of sugammadex or neostigmine to recovery of the TOF ratio to 0.8 and 0.7, and clinical signs of recovery assessed before transfer to the recovery room and before discharge from the recovery room (as described above).

Efficacy analyses were based on the intent-to-treat population, which included all the patients who received study drug and had at least one efficacy assessment. The all-subjects-treated group, which consisted of all the subjects who were randomized, and received a dose of study medication, was used for the safety analysis.

In order to include all patients from the intent-to-treat population, missing data for the primary parameter of time to a TOF ratio of 0.9 were imputed. For imputation of missing times from the start of administration of study drug to recovery of the TOF ratio to 0.7, 0.8, and/or 0.9, a conservative approach towards sugammadex was applied (described in detail elsewhere) [5]. For example, when time to a TOF ratio of 0.8 was available, the difference between the time to recovery of the TOF ratio to 0.8 and 0.9 for all patients randomized to sugammadex with available data was calculated. The 95th percentile of these differences was then added to the time to recovery of the TOF ratio to 0.8 for those patients with missing times to recovery of the TOF ratio to 0.9. The same method was used for the neostigmine group, but available data from patients randomized to neostigmine were included and the fifth percentile of the differences in time to recovery of the TOF ratio to 0.8 and 0.9 was used to impute missing data.

An interim analysis of efficacy was performed when 10 patients from each group had completed the study and provided data. The Hwang-Shih-de Cani method was used to statistically evaluate the primary efficacy parameter using validated data for the intent-to-treat population, with imputed data utilized in the case of missing values [7]. The interim analysis was conducted at a significance level of 0.0025 (one-sided), and the results analysis assessed by a Data and Safety Monitoring Board who were to make a recommendation to stop the neostigmine arm early if there were marked differences in efficacy between treatment arms. Enrollment continued into both groups during the data analysis and deliberations of the Data and Safety Monitoring Board. Because of the interim analysis, the primary analysis of the study was performed at a significance level of 0.0467.

## Results

### Patients

**Figure 1:**
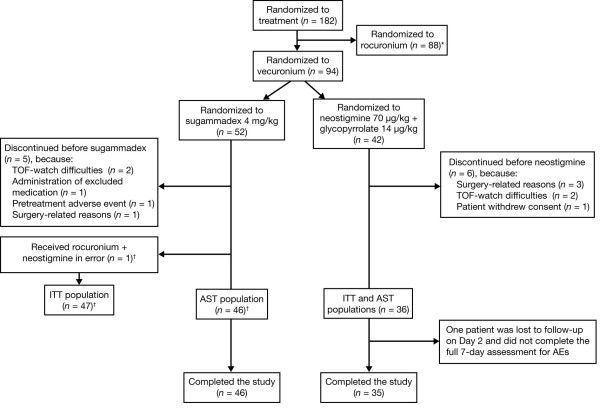
**Patient disposition**. One patient randomized to sugammadex/vecuronium was mistakenly given neostigmine/rocuronium and excluded from the AST population, but included in the ITT population according to the proposed randomization.*Data for patients randomized to the rocuronium arm have been reported elsewhere [5]. AST, all-subjects-treated; ITT, intent-to-treat; TOF, train-of-four.

A total of 94 patients (52 to sugammadex and 42 to neostigmine) were randomized at eight centers in the US (Stanford University Medical Center, Stanford, California; Advocate Illinois Masonic Medical Center, Chicago, Illinois; Vanderbilt University Medical Center, Nashville, Tennessee; Duke University Medical Center, Durham, North Carolina; Saddleback Memorial Medical Center, Laguna Hills, California; The State University of New York at Stony Brook, Health Sciences Center, Stony Brook, New York; Mayo Clinic, St Luke's Hospital, Jacksonville, Florida; University of California, San Francisco, Moffitt/Long Hospital and Mount Zion Hospital, San Francisco, California). After interim analysis, and recommendation by the Data and Safety Monitoring Board, the neostigmine group was discontinued because of marked differences in efficacy between treatments, although by this time 42 patients had already been randomized into the neostigmine group. A total of 11 patients (five sugammadex and six neostigmine) discontinued the trial before receiving the study drug. In addition, one patient randomized to vecuronium and sugammadex received rocuronium plus neostigmine and was excluded from the all-subjects-treated population, but was included in the intent-to-treat population according to randomization schedule (Figure 1). Therefore, the all-subjects-treated population consisted of 46 patients treated with sugammadex and 36 patients treated with neostigmine, and the intent-to-treat population consisted of 47 patients randomized to sugammadex and 36 patients randomized to neostigmine. Patients underwent varied surgical procedures, with the most common being gynecologic (37.8%), urologic (31.7%), open abdominal (9.8%), and laparoscopic abdominal (9.8%). Baseline characteristics were comparable between groups, except that the sugammadex group included more women (63% versus 42%), more patients who were American Society of Anesthesiologists class 1-2 (87% versus 64%), and had a younger mean age (50 versus 57 years) (Table 1).

**Table 1:** Baseline characteristics (all-subjects-treated population [n = 82])

	Sugammadex (n = 46)	Neostigmine (n = 36)
Gender		
Female, n (%)	29 (63)	15 (42)
Male, n (%)	17 (37)	21 (58)
Mean (SD) age, years	50 (14)	57 (12)
Mean (SD) weight, kg	86 (19)	86 (18)
Mean (SD) height, cm	170 (11)	173 (10)
Race, n (%)		
Asian	1 (2)	3 (8)
Black (of African heritage)	6 (13)	1 (3)
Caucasian	38 (83)	32 (89)
Other	1 (2)	0
ASA class, n (%)		
1	6 (13)	1 (3)
2	34 (74)	22 (61)
3	6 (13)	13 (36)
4	0	0

ASA, American Society of Anesthesiologists.

### Anesthesia and neuromuscular block

Generally, the groups were similar in the anesthetic agents administered. In total, 91% of sugammadex patients and 94% of neostigmine patients received sevoflurane maintenance anesthesia, with most patients receiving a range of sevoflurane concentrations over the course of anesthesia. In the sugammadex group as a whole, the sevoflurane end-tidal concentration ranged overall from 0.5-4.2% in the period before sugammadex administration and from 2.4-0.2% in the period after sugammadex administration. The corresponding concentrations in the neostigmine group overall were 0.3-4.0% before and 3.5-0.1% after neostigmine. The duration of sevoflurane exposure in the sugammadex group ranged from 14 minutes to 6.0 hours before sugammadex, and from 1 minute to 3.9 hours after sugammadex. The durations of sevoflurane exposure in the neostigmine group ranged from 45 minutes to 3.8 hours before and from 17 minutes to 5.2 hours after neostigmine. Four patients in the sugammadex group did not receive sevoflurane; three of these received isoflurane rather than sevoflurane (concentration ranging from 0.7-4.0% overall) and one patient received no maintenance anesthesia. In addition, in one sugammadex patient, sevoflurane was stopped after 14 minutes and desflurane (6-6.5%) administered instead. Two patients in the neostigmine group did not receive sevoflurane. They received isoflurane (0.3-0.9%) or desflurane (3.7-8.0%) instead. The inhalation anesthetic was maintained at least until recovery of the TOF ratio to 0.9 in 75% of patients in the two groups (determined in those patients in whom the time to a TOF ratio of 0.9 was known and the time of anesthetic discontinuation was recorded [n = 64]). Nitrous oxide was administered to 52% of patients in the sugammadex group and 56% of patients in the neostigmine group.

Forty-two sugammadex- and 32 neostigmine-treated patients received at least one maintenance dose of vecuronium. In patients receiving maintenance doses, a median of four (range 1-20) maintenance doses were administered in the sugammadex group and a median of two (range 1-12) maintenance doses were administered in the neostigmine group. In accordance with the protocol, in most patients (83% in the sugammadex group and 75% in the neostigmine group), the reversal drug was administered at a PTC of 1 or 2 after the last dose of vecuronium, although 7 patients in the sugammadex group and 9 patients in the neostigmine group received reversal at a higher PTC, including one patient who received neostigmine at a PTC of 6, which was considered to be a protocol violation. The median (range) time between administration of the last dose of vecuronium and administration of the reversal agent was 19.7 (3.4 to 102.7) minutes in the sugammadex group and 15.5 (3.2 to 112.4) minutes in the neostigmine group.

### Time to recovery

Four subjects in the sugammadex intent-to-treat group (including the patient who received rocuronium and neostigmine in error), and 19 in the neostigmine group had missing data for time to recovery of the TOF ratio to 0.9. In three of the sugammadex patients and ten of the neostigmine patients, the TOF-Watch had to be switched off before this level of recovery was reached; in one sugammadex patient, the time to TOF 0.9 was omitted from the assessments; and in the remaining nine neostigmine patients, the patient was moving or awake before a TOF ratio of 0.9 was reached. In addition, the Central Independent Adjudication Committee considered the time to the TOF ratio of 0.9 to be unreliable in two subjects from each group because of interference, an unstable recording or because of an incorrect set-up procedure of the TOF-Watch. Thus recovery data were imputed for these patients.

**Figure 2:**
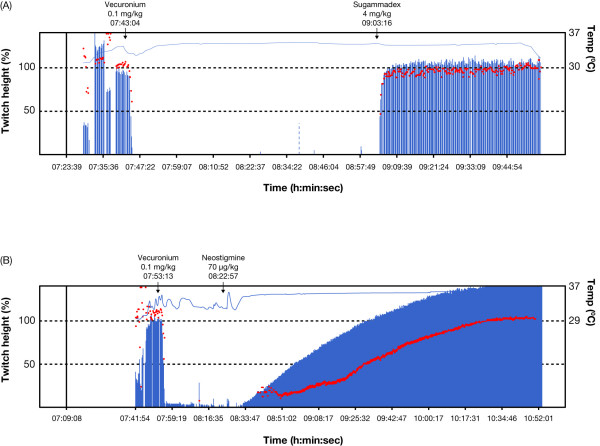
**Examples of recovery profiles for vecuronium 0.1 mg/kg after administration of (A) sugammadex 4 mg/kg or (B) neostigmine 70 μg/kg at a target of 1-2 PTC**. Bars represent first twitch (T_1_) values (twitch height %) and dots represent the TOF ratio. PTC, post-tetanic-counts; TOF, train-of-four.

**Figure 3:**
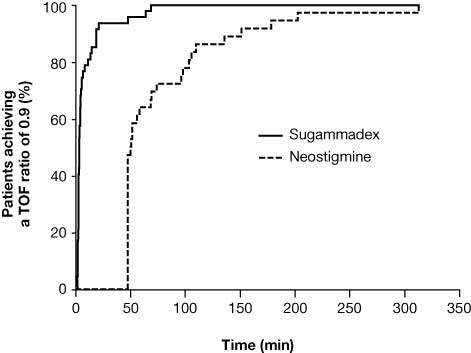
**Time (min) from start of administration of sugammadex or neostigmine to recovery of the TOF ratio to 0.9 (intent-to-treat population, imputed data, *n *= 47 for sugammadex and *n *= 36 for neostigmine)**. TOF, train-of-four.

Figure 2 shows representative examples of the recovery profile from neuromuscular block after sugammadex and neostigmine administration. Geometric mean time to recovery of the TOF ratio to 0.9 was significantly faster with sugammadex compared with neostigmine (4.5 minutes versus 66.2 minutes, p < 0.0001) (Table 2). The median (range [interquartile range] time to recovery of the TOF ratio to 0.9 was 3.3 (1.4-68.4 [2.3-6.6]) minutes in the sugammadex group versus 49.9 (46.0-312.7 [46.0-96.6]) in the neostigmine group. The faster time to recovery in the sugammadex group is also shown in Figure 3, which compares the cumulative percentage of patients who recovered to a TOF ratio of 0.9 over the course of the study in sugammadex and neostigmine groups, versus time after administration of the study drug. In a separate analysis including only those patients with data available for the time to TOF 0.9 (n = 41 in the sugammadex group and n = 15 in the neostigmine group), the geometric mean recovery times were 3.6 minutes versus 91 minutes, respectively (p < 0.0001).

**Table 2:** Time to recovery of the train-of-four ratio to 0.9, 0.8, and 0.7.

	Sugammadex (n = 47)^†^	Neostigmine (n = 36)
Time to TOF ratio of 0.9, min		
Geometric mean	4.5*	66.2
Median	3.3	49.9
Interquartile range	2.3-6.6	46.0-96.6
Range	1.4-68.4	46.0-312.7
Time to TOF ratio of 0.8, min		
Geometric mean	3.3*	58.9
Median	2.7	43.9
Interquartile range	1.8-4.4	42.9-79.8
Range	1.2-65.2	35.3-250.9
Time to TOF ratio of 0.7, min		
Geometric mean	2.6*	48.8
Median	2.5	36.4
Interquartile range	1.6-3.3	34.9-67.5
Range	1.1-61.9	27.5-192.2

Recovery times were measured from the start of administration of sugammadex or neostigmine. Data are given for the intent-to-treat population, with imputed data.

*p < 0.0001 *versus *neostigmine. ^†^Data include the patient who received rocuronium plus neostigmine reversal rather than vecuronium plus sugammadex reversal in error, the times to a TOF ratio of 0.9 and 0.8 were not available. Her times to TOF 0.9 and 0.8 were imputed based on her time to TOF 0.7 of 61.9 minutes.TOF, train-of-four.

The geometric mean times to recovery of the TOF ratio to 0.7 and 0.8 were also significantly faster with sugammadex compared with neostigmine (p < 0.0001) (Table 2). The patient who was randomized to vecuronium and sugammadex but received rocuronium and neostigmine in error had no data available for the times to a TOF ratio of 0.8 and 0.9. Her time from study drug administration to a TOF ratio of 0.7 was 61.9 minutes and her imputed times to a TOF ratio of 0.8 and 0.9 were 65.2 and 68.4 minutes, respectively. Thus, these were the longest times to recovery in each case (Table 2). Two other patients in the sugammadex group also had relatively long times to a TOF ratio of 0.9 of 47.4 and 63.5 minutes. In these patients, the times to a TOF ratio of 0.7 were 2.9 and 7.3 minutes, respectively. In the first of these two patients, the investigator reported difficulties in calibrating, and commencement of tracing prior to adequate baseline TOF values being established.

In the sugammadex group, most patients (n = 44) received an intubating dose plus one or more maintenance doses of vecuronium. Geometric mean time to reversal of vecuronium-induced block appeared to be similar in patients who received at least one maintenance dose of vecuronium compared with those patients who received an intubating dose only (4.5 versus 4.2 minutes).

### Clinical signs of recovery

Except for nine patients in the sugammadex group and 10 patients in the neostigmine group, the majority of patients were considered cooperative before transfer to the recovery room, allowing the 5-second head-lift and general muscle weakness tests to be performed. In those patients with assessments before discharge from the recovery room (n = 41 in the sugammadex group and n = 34 in the neostigmine group), all except two patients in the sugammadex group and one in the neostigmine group were awake and oriented, all patients were considered cooperative, and all patients, except for one in the neostigmine group, were able to perform the 5-second head-lift. Of the 41 sugammadex patients and 34 neostigmine patients with assessments, four patients showed signs of mild general muscle weakness, as assessed by the general muscle weakness scale, before discharge from the recovery room, (sugammadex, n = 1 [score of 8]; neostigmine, n = 3 [scores of 7, 8, and 8]) but all affected patients could perform the 5-second head-lift test before discharge from the recovery room.

### Safety assessments

In the all-subjects-treated group, all patients in the sugammadex group and 33 patients in the neostigmine group (92%) had at least one AE. Table 3 shows AEs occurring in ≥10% of patients in either treatment group, regardless of relationship to study drug. Most AEs were of mild-to-moderate intensity. The most commonly reported AEs in both groups were procedural pain and nausea.

**Table 3:** Adverse events occurring in at least 10% of patients in either treatment group.

Adverse event	Sugammadex (n = 46)	Neostigmine (n = 36)
Procedural pain	33 (71.7)	24 (66.7)
Nausea	24 (52.2)	12 (33.3)
Incision-site complication	10 (21.7)	8 (22.2)
Pharyngolaryngeal pain	8 (17.4)	7 (19.4)
Headache	12 (26.1)	2 (5.6)
Vomiting	9 (19.6)	4 (11.1)
Dizziness	5 (10.9)	4 (11.1)
Pruritus	5 (10.9)	2 (5.6)
Post-procedural nausea	5 (10.9)	2 (5.6)
Constipation	5 (10.9)	0
Chills	5 (10.9)	0

Adverse events are listed regardless of their perceived relationship to study drug for the all-subjects-treated population (n = 82).

Nine patients (19.6%) in the sugammadex group experienced a total of 18 drug-related AEs and 10 patients (27.8%) in the neostigmine group experienced a total of 21 drug-related AEs. The most commonly reported drug-related AEs were nausea or post-procedural nausea (sugammadex, n = 5; neostigmine, n = 3) and leukocytosis (sugammadex, n = 1; neostigmine, n = 2). All of the other drug-related AEs in both groups were isolated reports and most were of mild (16/18 in the sugammadex group; 8/21 in the neostigmine group) or moderate (2/18 in the sugammadex group; 10/21 in the neostigmine group) intensity. There were only three severe AEs (anxiety, depression, and fatigue) experienced by a patient in the neostigmine group and considered by the investigator to be possibly drug related. There were no deaths or serious drug-related AEs in either treatment group.

Laboratory parameters and vital signs were similar between groups, with the exception of an increase in mean heart rate from baseline in the neostigmine group 2-10 minutes post dose. However, there were no clinically significant differences between the groups in terms of the percentage of patients with markedly abnormal heart rate values at any of the assessments, and mean heart rate in the neostigmine group returned to baseline level at subsequent assessment points. There was no clinical evidence of residual neuromuscular block or reoccurrence of neuromuscular block either clinically (respiratory problems) or according to study neuromuscular transmission guidelines (significant decrease in the TOF ratio to <0.8) in either group.

## Discussion

This is the first active-controlled study to show that sugammadex effectively reverses profound vecuronium-induced block. Recovery from neuromuscular block induced by vecuronium was almost 15-fold faster with sugammadex 4 mg/kg than with neostigmine 70 μg/kg when given at a target of 1-2 PTCs.

There were several missing times to recovery of the TOF ratio to 0.9 in the study, especially in the neostigmine group (19 missing recovery times compared with 4 missing recovery times in the sugammadex group) resulting from for example patients waking up and moving so that the TOF trace was not completed. We overcame the issue of missing recovery times by using a conservative imputation technique for the primary analysis, where missing recovery times were estimated using a worst-case scenario for patients receiving sugammadex and a best-case scenario for patients receiving neostigmine. The geometric mean time to a TOF ratio of 0.9 was 3.6 minutes in the sugammadex group versus 91 minutes in the neostigmine group in an analysis including only those patients with a recorded time to TOF 0.9 (n = 41 in the sugammadex group and n = 15 in the neostigmine group). Thus, using both the conservative imputation technique and the observed cases only, a considerably faster time to recovery was seen with sugammadex 4 mg/kg versus neostigmine at 1-2 PTCs.

One other study, a dose-finding study, has investigated the efficacy of sugammadex administered during profound vecuronium-induced block (at 1-2 PTCs) [3]. This study showed a mean recovery time of 3.3 minutes (n = 8) with sugammadex 4 mg/kg [3], consistent with the current study. Previous reports of the efficacy of sugammadex in rocuronium-induced profound block have shown recovery to a TOF ratio of 0.9 in 2-3 minutes [4,5].

In the current study, when considering only those patients in whom times to TOF 0.9 were available, 80% had recovered within 5 minutes of sugammadex administration. In contrast, no neostigmine patients had recovered within 5 min, and 36% did not recover to a TOF ratio of 0.9 until >60 minutes after administration of neostigmine (Figure 3). Three patients receiving sugammadex had a measured (rather than imputed) time to a TOF ratio of 0.9 of >15 minutes. Time to reversal in these patients was 18.7, 47.4 and 63.5 minutes, respectively. This may reflect technical difficulties with the TOF-Watch as calibration data were not adequate prior to start of monitoring, with unstable individual twitch responses together with unstable and fluctuating TOF ratio values. At recovery, significant scatter was observed as unstable twitch responses and unstable TOF ratio values.

To optimize the reversal achieved with acetylcholinesterase inhibitors, some spontaneous recovery of neuromuscular function is required before administration [8], and our study confirms this. This means that clinicians may be unable to maintain profound block to the end of surgery without the penalty of the time required for spontaneous recovery prior to acetylcholinesterase inhibitor administration, as well as the fear of residual block in the recovery room in a patient who has not adequately recovered from the block. As shown in the current study, neostigmine is not effective for the rapid reversal of profound vecuronium-induced neuromuscular block, with the median time to a TOF ratio of 0.9 of 49.9 minutes and, in fact, one patient took >5 hours to achieve this level of recovery. Furthermore, 75% of neostigmine patients recovered to a TOF ratio of 0.9 in ≤96.6 min while, in the sugammadex group, 75% achieved the same level of recovery in ≤6.6 min. This suggests increased predictability of reversal of vecuronium-induced NMB with sugammadex compared with neostigmine.

It is known that, even with recovery of the TOF ratio to 0.9 or more, muscle weakness from impaired neuromuscular transmission can occur [9]. In addition to using the TOF-Watch, neuromuscular recovery was assessed using clinical tests (5-sec head-lift test and a test for general weakness). The 5-second head-lift test may be considered insensitive [10] and inappropriate for the detection of residual block [11], and the test for general muscle weakness has not been formally validated and was intended more as a clinical measurement tool for overall well being. However, these tests were not meant to stand alone but rather to complement the findings of the objective neuromuscular monitoring. On transfer from the recovery room, all except 2% of patients in the sugammadex group and 9% in the neostigmine group who underwent these tests had no evidence of muscle weakness and all but one patient in the neostigmine group could perform the 5-second head-lift test.

Inhalational anesthetics such as sevoflurane and isoflurane can significantly prolong the duration of action of rocuronium and vecuronium [12,13], and it is well known that volatile anesthetics can also prolong the time to reversal with acetylcholinesterase inhibitors [2]. Most of the patients in the current study received maintenance anesthesia with sevoflurane, and only one patient received no inhalation anesthetics. We showed that sugammadex reversal of profound block was rapid even in the presence of sevoflurane, isoflurane or desflurane. These findings are in agreement with previous studies, which show sugammadex to be equally effective at reversing rocuronium-induced block under maintenance anesthesia with sevoflurane or propofol [14,15].

Acetylcholinesterase inhibitors are associated with various adverse effects related to the increase they cause in acetylcholine concentration at *muscarinic *receptors (in addition to the nicotinic receptors as their intended site of action), therefore co-administration with an anticholinergic agent such as glycopyrrolate is often required. By virtue of its mode of action in encapsulating rocuronium and vecuronium molecules, sugammadex was anticipated to be well tolerated [16]. Although our study was underpowered for many of the safety related issues, sugammadex was well tolerated, both in the vecuronium arms described here and in the rocuronium arms of the study [5]. The most commonly reported AEs in both groups were procedural pain and nausea. More patients in the sugammadex group experienced headache, nausea, or vomiting compared with the neostigmine group, although most of these occurrences were not considered by the investigator to be related to the study drug. In total, 20% of sugammadex-treated patients and 28% of neostigmine-treated patients experienced AEs that were considered possibly, probably, or definitely related to study drug. Apart from nausea, most of these were isolated reports and of mild-to-moderate intensity. There were no serious drug-related AEs in any treatment group. Many of the tests available for the determination of mild residual paralysis or reoccurrence of block in the awake patient have shortcomings. Bearing in mind this limitation, there was no evidence of residual paralysis or reoccurrence of block found for any patient in this study.

## Conclusions

In conclusion, sugammadex provided effective and rapid reversal of profound neuromuscular block induced by vecuronium under sevoflurane anesthesia.

## Competing interests

This study was funded by Merck Research Laboratories, Summit, New Jersey, US. Hendrikus Lemmens has participated in a Merck advisory board. Jovino Ben Morte is an employee of Merck Research Laboratories, Summit, New Jersey, US. Mohammad El-Orbany has received research funding from Merck. James Berry and Gavin Martin declare that they have no other competing interests.

## Authors' contributions

HL, MEl-O, JB and GM were involved in the conduct of the study and data collection. JM was involved in the study design and data analysis. All authors have read and approved the final manuscript

## Pre-publication history

The pre-publication history for this paper can be accessed here:

http://www.biomedcentral.com/1471-2253/10/15/prepub
